# Research progress on molecular mechanisms of general anesthetic-induced neurotoxicity and cognitive impairment in the developing brain

**DOI:** 10.3389/fneur.2022.1065976

**Published:** 2022-11-24

**Authors:** Jiaojiao Wang, Zhihui Liu

**Affiliations:** ^1^Department of Anesthesiology, Baotou Central Hospital, Baotou, China; ^2^Baotou Clinical Medical College, Inner Mongolia Medical University, Baotou, China

**Keywords:** general anesthetics, neurotoxicity, cognitive impairment, molecular mechanisms, developing brain

## Abstract

General anesthetics-induced neurotoxicity and cognitive impairment in developing brains have become one of the current research hotspots in the medical science community. The underlying mechanisms are complex and involve various related molecular signaling pathways, cell mediators, autophagy, and other pathological processes. However, few drugs can be directly used to treat neurotoxicity and cognitive impairment caused by general anesthetics in clinical practice. This article reviews the molecular mechanism of general anesthesia-induced neurotoxicity and cognitive impairment in the neonatal brain after surgery in the hope of providing critical references for the treatments of clinical diseases.

## Introduction

There is increasing evidence that exposure to general anesthesia in early life can lead to apoptosis of developing nerve cells, which may eventually develop into cognitive dysfunction (1). Infants and young children at critical stages of brain development are at significantly increased risk of postoperative neurotoxicity and cognitive impairment when using general anesthetics for surgical procedures. How to reduce and deal with this risk is an important issue faced by clinicians. The key to solving this problem is to clarify the related mechanisms of general anesthetics-induced neurotoxicity and cognitive impairment in the developing brain. This article reviews the research progress on the molecular mechanisms of general anesthetics-induced neurotoxicity and cognitive impairment. A summary of relevant evidence studies is presented in Table 1.

**Table 1:** Related studies on neurotoxicity of common general anesthetics.

**General anesthetics**	**Related molecular studies**
**Inhalation anesthetics**	
Halothane	Increased inflammatory reaction of brain (91)
Nitrous oxide	Vitamin B_12_ deficiency (92, 93)
Sevoflurane	Iron overload (94, 95), Decreased number of excitatory synapses and protein levels (14, 31)
Isoflurane	Ferroptosis (96, 97), Neuronal cell cycle activation (98), Migration of dentate gyrus granule cells (99, 100)
Desflurane	Decrease synaptic integrity (101), Decreased NMDAR-mediated excitatory postsynaptic current (102)
**Intravenous anesthetics**	
Ketamine	Iron overload (95), Increased NMDAR at extrasynaptic sites (103–105)
Propofol	Altered synaptic plasticity (106, 107), Mitochondrial damage in hippocampal neurons (108)

## Related signaling pathways

### HIPK2/Akt/mTOR signaling pathway

Homeodomain interacting protein kinase 2 (HIPK2), protein kinase B (PKB/ Akt), mammalian target of rapamycin (mTOR), are serine-threonine protein kinases, which can be activated or inhibited by growth factors, cellular energy status, and nutrients through substrate phosphorylation and are involved in apoptosis, autophagy, and synaptic plasticity. Meanwhile, it may be related to the mechanism of neurotoxicity of the developing brain induced by general anesthetics. General anesthetics can promote the apoptosis of neural cells in the newborn brain by activating HIPK2 and promoting its expression.

It has been reported that upregulation of HIPK2, Akt, and mTOR signaling can be detected in apoptotic neurons in mice exposed to sevoflurane, and that inhibition of this signaling promotes neuronal apoptosis in the hippocampus of newborn brains, protects developing hippocampal neurons, and reduces their apoptosis (2). Because HIPK2 itself can promote cell apoptosis, activating HIPK2 can promote the activation of the Akt/mTOR signaling pathway that inhibits neuronal apoptosis, which may be caused by negative feedback regulation of the body, and this feedback mechanism needs further verification by researchers. Xu et al. found that the up-regulation of mTOR is not only found in sevoflurane-induced neuronal cells but also in the induction of other general anesthetics such as isoflurane and propofol (3, 4). The mechanisms by which the HIPK2/Akt/mTOR pathway contributes to neurotoxicity of developing brains are likely to occur in all commonly used general anesthetics, and in the last few years, new advances have been made in the study of their functions and regulatory mechanisms, but the importance of these signals and the background mechanisms remain to be confirmed, which points out the direction for medical research and requires further exploration of medical research.

### PI3K/Akt signaling pathway

As a proto-oncogene, Akt can regulate various cellular functions, including several processes such as cell metabolism, growth, proliferation, survival, and transcription. As a factor capable of activating the amplification of the Akt signaling cascade, Phosphatidylinositol 3-kinase (PI3K) is an intracellular phosphoinositide kinase. When the upstream signal comes, it couples to Akt through the interaction between subunits and then activates its downstream signaling molecule, mTOR, to inhibit apoptosis (5). Akt can deregulate cell survival through pro-apoptotic signals that directly interfere with the production of transcription factors, such as Forkhead box O (FoxOs). Studies have shown that the Akt/FoxO1 signaling pathway mediates sevoflurane-induced neuronal apoptosis in neonatal mice (6), thereby, activating this pathway can inhibit apoptosis of neuronal cells in neonatal brains (7, 8). In addition, activation of the PI3K/Akt/mTOR signaling pathway can improve general anesthetic-induced neuroinflammation in rats and reduce the neurotoxic effect of general anesthetics (9, 10). Nowadays, researches on the early potential treatment of the PI3K/Akt signaling pathway by general anesthetics for their neurotoxic effects in the developing brain have been gradually unfolded. Studies have found that both Panax Notoginseng Saponins and Hemin can inhibit neuronal apoptosis by activating the Akt signaling pathway (11, 12), which in turn attenuates the neurotoxicity and cognitive impairment caused by general anesthetics. In addition, Atractylenolide III has also been shown to produce the same benefits in the same way, that is, activated the PI3K/Akt/mTOR signaling pathway, which can inhibit neuronal apoptosis (13). It follows that drugs targeting this signaling pathway could be useful in treating the neurotoxicity that results from general anesthesia in the newborn brain.

### HIPK2/JNKs/c-Jun signaling pathway

c-Jun N-terminal kinases (JNKs) belong to the mitogen-activated protein kinase family (MAPK) and are involved in apoptosis. The study found that JNKs and their downstream c-Jun were involved in the neurotoxicity of sevoflurane in the neonatal brain; meanwhile, up-regulation of HIPK2, JNK, and c-Jun was observed in hippocampal neurons of mice exposed to sevoflurane, continued until adulthood (14), inhibition of JNK found that mice can restore normal neurodevelopment and cognitive function (14, 15), but activation of this signaling pathway can induce neuronal apoptosis (16), which may be linked to its upregulation of Connexin-43, and its increase can lead to cognitive dysfunction (17). And when JNK antagonists were given to these newborn mice, it was found that the expression of HIPK2 was not affected (14), so JNKs/c-Jun signaling pathway may be directly induced by general anesthetics, but not related to the presence of HIPK2. That is to say, as a tumor suppressor gene, HIPK2 can increase independently and lead to neuronal apoptosis when stimulated by general anesthetics, and play a promoting role in the JNKs/c-Jun signaling pathway. Therefore, inhibition of the HIPK2/JNK/c-Jun signaling pathway has specific guiding significance for treating neonatal brain neurotoxicity and cognitive impairment, and it can be used as a potential target to treat general anesthetic-induced neurotoxicity and cognitive impairment. In addition, this pathway may also be involved in isoflurane-induced apoptosis of neonatal rat hippocampal neurons (18, 19). However, whether it is involved in other mechanisms of general anesthetic neurotoxicity requires further confirmation.

### JAK/STAT signaling pathway

Interferon, interleukin, growth factors, and other chemical messengers activate Janus Kinase (JAK) and phosphorylate it, and combine with downstream transcription factor that is signal transducer and activator of transcription (STAT) to initiate transcription, which in turn affects cell proliferation, differentiation, inflammation, and so on. Studies have found that sevoflurane may induce neurotoxicity, particularly in response to damage to the astrocytes in the hippocampus of neonatal rats by inactivating the JAK/STAT signaling pathway (20). Sevoflurane exposure can lead to an increase in the release of proinflammatory factors (21), which in turn leads to widespread inflammation in the brain, and targeting the activation of the JAK/STAT signaling pathway can effectively inhibit the occurrence of inflammation in the nervous system (22–24), thus promoting the expression of this pathway or inhibiting its inactivation has an important guiding significance for the treatment of neurotoxicity caused by general anesthetics.

### AMPK signaling pathway

Activation of adenylate-activated protein kinase (AMPK) often appears as a heterotrimeric complex, and its activation may be induced by the inhibition of respiration and hemodynamic effects of general anesthetics, after which it is involved in regulating processes such as cell proliferation and organismal metabolism by activating downstream signals (such as FoxO3a, Nrf2, etc.). Studies have found that AMPK affects the activation status of microglia and neuronal cell survival in the adult and developing brain (25), and activation of the AMPK signaling pathway may inhibit the occurrence of neuroinflammation (26, 27) and play a neuroprotective role. In addition, Zhang et al. found that sevoflurane-induced neuronal apoptosis and hindered proliferation are related to the inactivation of the AMPK/FoxO3a signaling pathway (28), so up-regulation of this signaling pathway may attenuate this neurotoxicity and play a neuroprotective effect. Dai et al. found that sevoflurane-induced microglial inflammatory injury and neurotoxicity were alleviated by activating (29). Thus, up-regulation of the AMPK signaling pathway may become a potential therapeutic approach for the current treatment of general anesthetics.

In conclusion, the activation of the Akt/mTOR signaling pathway, MAPK signaling pathway, and JAK/STAT signaling pathway can inhibit the apoptosis of nerve cells or glial cells. When general anesthetics act on the newborn brain, their toxic effects on the brain, including apoptosis of cells in the nervous system, cerebral ischemia, and hypoxia, will directly or indirectly activate these signaling pathways in feedback, thereby reducing brain damage. Although this feedback needs further scientific proof, many studies have detected the activation of the above signaling pathways in the newborn brain exposed to general anesthetics. It seems that although general anesthetics can cause neurotoxicity in the infant's brain, the body has a self-repairing function, which also verifies that long-term exposure to anesthetics can seriously damage the development of the infant's brain. However, a short time of exposure to general anesthetics will not cause significant damage (30, 31).

## Non-coding RNA

### MiRNA

MicroRNA (miRNA) is a type of non-coding single stranded RNA, which can specifically recognize targeted mRNA, degrade it and inhibit its function. It has been found that many miRNAs can participate in the process of neuronal proliferation and apoptosis by regulating the proteins in the signaling pathway, especially the Akt signaling pathway that can inhibit neuronal apoptosis. Liu et al. found that miR-204-5p can mediate the neurotoxicity of the hippocampus of newborn rats caused by general anesthetics by targeting brain-derived neurotrophic factor (BDNF), and its down-regulation can inhibit neuronal apoptosis, thereby reducing cytotoxicity, which may be related to the up-regulated BDNF/Akt signaling pathway (32). Similarly, Wang et al. found that in sevoflurane-exposed mice, up-regulation of miR-1297 expression enhanced the expression of phosphatase and tensin homolog deleted on chromosome ten, which in turn stimulated sevoflurane-induced neurotoxicity through inhibiting the Akt signaling pathway (33), leading to neuronal apoptosis and cognitive impairment. Therefore, inhibiting the expression or promoting the degradation of miR-204-5p and miR-1297 may be a potential therapeutic method for treating general anesthetic-induced neurotoxicity and cognitive impairment in the developing brain by indirectly upregulating the Akt signaling pathway, which can inhibit neuronal apoptosis. At present, great progress has been made in the treatment of neurotoxicity of general anesthetics by targeting miRNAs. Recent studies have shown that miR-17-5P can alleviate the neurotoxicity induced by propofol and isoflurane exposure (34, 35), which may serve as a potential drug direction for future research and development of general anesthetic neurotoxicity. Similarly, Chen et al. found that miR-384-3p can alleviate sevoflurane-induced apoptosis and morphological changes of hippocampal neurons, and also improve cognitive impairment, such as the sevoflurane-induced decline in learning and memory ability (36), which is of great help in relieving and treating cognitive impairment caused by general anesthesia and is a potential new therapeutic strategy. Other studies found that down-regulation of miR-27a-3p (37), miR-494-3p (38), and miR-183 (39) could protect nerve cells and ameliorate general anesthetic-induced neurotoxicity and cognitive impairment. All of them may serve as potential therapeutic targets for neurotoxicity in the developing brain in the future.

### LncRNA

LncRNA belongs to a class of non-coding RNA, most of which are specific in expression patterns, that is, different expression patterns are involved in neural cell differentiation, proliferation, and apoptosis through different pathways. At present, lncRNA has been proven to be the critical mediator of neurotoxicity. Similar to miRNAs, the mechanism leading to neurotoxicity in the developing brain is related to regulation of signaling pathways involved in neuronal apoptosis. Studies have found that lncRNA may promote the apoptosis of hippocampal neurons in the developing brain (40). It was found that lncRNA Neat1 upregulates Serine-threonine protein kinase through spongy miR-298-5p, which ultimately leads to general anesthetic-induced neurotoxicity (41). At the same time, abnormally expressed lncRNA may mediate the apoptosis of newborn brain neurons and cognitive dysfunction by general anesthetics (42). These findings may provide new ideas for the treatment of general anesthetic neurotoxicity in the developing brain. There is also long non-coding RNA metastasis-associated lung adenocarcinoma transcript 1 (lncRNA MALAT1), which can promote neurogenesis and exert brain protection by regulating the MAPK signaling pathway. Experiments have shown that inhibition of MALAT1 can reduce the apoptosis of hippocampal neurons caused by anesthetics in newborn mice (43, 44), and improve learning ability and memory function (45), which may be related to the down-regulation of MALAT1, which can activate PI3K/Akt signaling pathway that can inhibit neuronal apoptosis. In addition, Lu et al. found that the expression of the lncRNA Gadd45a gene in mouse neurons exposed to sevoflurane increased (46), which may be related to sevoflurane-induced neurotoxicity, which revealed a new target for the molecular mechanism of sevoflurane-induced neurotoxicity point. In summary, lncRNAs are involved in general anesthetic-induced neurotoxicity and cognitive impairment through various expression patterns. However, there is no apparent progress in the treatment of lncRNA-related targets, and further exploration by researchers is needed.

### CircRNA

Circular RNAs (circRNAs) are vital components in the regulation of neuronal gene expression in the brain, and the expression pattern of circRNAs changes stage by stage during the newborn brain development (47, 48), and dynamic changes in their expression are critical for brain function and maintenance of brain physiological homeostasis (49). General anesthetics can affect the expression of circRNA in the brain and nerve cells. Studies have found that ketamine can cause abnormal expression of circRNA in the hippocampus of rats, mainly showing changes in quantity and folding (50). Then the neurotoxicity induced by general anesthetics in the developing brain may be related to circRNA, some studies have shown that circRNA001372 inhibits propofol-induced neurotoxicity through activating PI3K/Akt signaling pathway in rat brain and neuronal cells (51). Although not investigated for the developing brain, there is still some guidance for the study of circRNA promoting signaling pathways that can inhibit neuronal apoptosis in the newborn brain. There are currently relatively few studies on the neurotoxicity and cognitive impairment produced by circRNA for general anesthetics to induce in the developing brain, which need further efforts by researchers to explore.

## Molecular protein

### Klotho protein

Klotho protein is a major component of the endocrine fibroblast growth factor receptor complex and has important protective effects on hippocampal neuronal cells. Studies have found that the Klotho protein is closely related to cell apoptosis and autophagy. Autophagy may be involved in general anesthetic-induced neurotoxicity, and studies have found that Klotho protein may play a protective role in neuronal cells by regulating autophagy and promoting protective autophagy by activating the PI3K/Akt signaling pathway, which may be a potential therapeutic approach for general anesthetic-induced neurotoxicity (52). Thus, the Klotho protein can protect neuronal cells through autophagy. Lianet al. found that Klotho protein can reduce sevoflurane-induced oxidative stress and mitochondrial damage in neonatal brain neurons (53). When sevoflurane is administered to neonatal rats, organismal stress leads to increased expression of Klotho protein and the mRNA encoding this protein in the hippocampus of neonatal rats, thereby alleviating the neurotoxic effects of sevoflurane in the neonatal brain (53). It has been shown that Klotho protein activates the transcription of the antioxidant enzyme manganese superoxide dismutase to relieve oxidative stress and reduce hippocampal neuronal injury (54), and whether this enzyme has a correlation with the PI3K/Akt signaling pathway needs to be further explored. In conclusion, upregulation of Klotho protein expression of this protein is very helpful for alleviating neurotoxicity and cognitive impairment in the newborn brain caused by general anesthetics, and Klotho protein may serve as a research direction for the treatment of general anesthetic-induced neurotoxicity and cognitive impairment in the neonatal brain.

### DJ-1

DJ-1 belongs to a member of the peptidase C56 family of proteins, which can exert neuroprotective effects through multiple pathways (55). Studies have shown that wild-type DJ-1 can inhibit sevoflurane-induced apoptosis by regulating the mitochondrial pathway, thereby protecting neuronal cells from general anesthetic-induced neurotoxicity. Still, when it mutates to DJ-1 (L166P), the neuroprotective effect is lost, and the neurotoxicity is exacerbated (56). Simultaneously inhibiting the expression of DJ-1 can aggravate isoflurane-induced neuronal apoptosis by regulating the mitochondria-dependent apoptosis pathway, and the feedback up-regulation of DJ-1 can reduce the neurotoxicity and cognitive impairment induced by isoflurane (57). Gestation is a critical period in the development of the pediatric brain, especially early in gestation, when cell numbers increase rapidly. Trophoblast cells provide nutrients for embryonic development. DJ-1 is mainly expressed in trophoblast cells in the placenta and its expression increases during the first trimester. DJ-1 has been found to maintain trophoblast function, possibly through the Akt signaling pathway, and to play a protective role in brain development during pediatric embryogenesis (58). Therefore, DJ-1 can be used as a potential biomarker for diagnosing general anesthetic-induced neurotoxicity and as a therapeutic target for the prevention and intervention of general anesthetic neurotoxicity and cognitive impairment in the developing brain.

### Apolipoprotein E and tau protein

Apolipoprotein E (ApoE) may mediate general anesthetic-induced neuronal toxicity and cognitive impairment in the developing brain. Neurotoxicity and cognitive impairment in mice exposed to sevoflurane may be related to the expression of ApoE and its toxic fragments. It has been found that ApoE toxic fragment (18-kDa fragment) promotes sevoflurane-induced Tau phosphorylation and neuroinflammation *in vitro* (59). Therefore, promoting the degradation of its toxic fragments or inhibiting their expression may be potential strategies for the treatment of general anesthetics-induced neurotoxicity and cognitive impairment in the developing brain. In addition, Tau is a microtubule-associated protein, and the hyperphosphorylated form of Tau is significantly increased after nerve injury (60), so it is considered to be an important factor affecting general anesthetics leading to neurotoxicity and cognitive impairment in the newborn brain. ApoE toxic fragment may be one of the potential mechanisms of neuronal Tau phosphorylation in neonatal mice exposed to sevoflurane (61). Coenzyme Q10 may reduce ApoE and phosphorylated Tau expression, thereby attenuating sevoflurane-induced neuroinflammation in mouse hippocampal neurons of newborn mice (62). Thus, targeting the regulation of Tau phosphorylation levels may also be a potential therapeutic direction for general anesthetics-induced neurotoxicity in the neonatal brain.

### Histone deacetylase 2

Histone deacetylase 2 (HDAC2) is a protein that regulates the transcription of memory-related genes. The study found that rats exposed to sevoflurane impaired cognitive functions such as learning and memory in offspring mice by upregulating HDAC2, and inhibition of HDAC2 attenuated these impairments (63). This suggests that HDAC2 may mediate the process of neurotoxicity and cognitive damage induced by general anesthetics in newborn brain cells, and it can reduce the neurotoxicity and cognitive damage induced by general anesthetics by inhibiting the expression of HDAC2. Lianget al. found that isoflurane exposure in neonatal mice caused cognitive impairment during puberty by reducing histone acetylation in the hippocampal glutamatergic system, which may be related to the upregulation of HDAC2 (64). However, administration of sodium butyrate (NaB) not only restored histone acetylation in hippocampal neurons but also improved cognitive impairment *in vivo* so NaB may be a potential therapeutic drug for isoflurane exposure-induced cognitive impairment (64). It was found that artemisinin reduced the expression of HDAC2 and HDAC3 and increased histone acetylation, and artemisinin could also effectively regulate isoflurane-induced JNK signal activation and down-regulate ERK1/2 expression (65). It seems that histone acetylation may have a negative correlation with JNK signal activation, but this needs further proof by researchers. Another study found that the decline of learning and memory ability caused by repeated exposure to isoflurane in neonatal mice is related to the dysregulation of histone acetylation in the hippocampus, and the promotion of histone acetylation and histone acetylation-mediated gene expression can improve this cognitive impairment (66). Therefore, promoting histone acetylation, inhibiting the production of HDAC2, or promoting its degradation can be potential strategies for the treatment of general anesthetic neurotoxicity and cognitive impairment in the developing brain.

### GABA_A_R and BDNF

Type A gamma-aminobutyric acid receptor (GABA_A_R) is the target receptor for sevoflurane (67). It contains two subtypes, GABA_A_R_α2_ and GABA_A_R_α1_. Studies have found that the ratio of α1/α2 subunits increases under sevoflurane exposure, so sevoflurane-induced neurotoxicity in the developing brain may be related to the transmission of GABA_A_R_α2_ to GABA_A_R_α1_ (68). From this point of view, by interfering with the transition between GABA_A_R_α2_ and GABA_A_R_α1_, finding an appropriate ratio between the two subtypes is of great significance for the prevention and treatment of anesthetic neurotoxicity and cognitive impairment in newborn children induced by general anesthetics. In addition, GABA_A_R has complex bidirectionality in early brain development. Its hyperactivation leads to the reduction of BDNF in the hippocampus and frontal cortex (69), which can cause neurological and cognitive impairment; Its inhibition can also affect synaptic plasticity and cognitive function and downregulate BDNF (70, 71). The study found that BDNF, as a protein with a neurotrophic effect, can improve sevoflurane anesthesia-mediated cognitive impairment of hippocampal neurons in aged rats (72), which may be related to the activation of the Akt signaling pathway (32). The down-regulation of BDNF may be related to the neurotoxicity induced by general anesthetics, it was found that multiple inhalations of sevoflurane in neonatal rats could inhibit the cleavage of proBDNF by disrupting the balance of the tissue plasminogen activator (tPA) and plasminogen activator inhibitor type 1 (PAI-1) fibrinolytic system, resulting in the down-regulation of BDNF, thereby blocking the activation of the downstream the Akt signaling pathway promotes hippocampal neuronal apoptosis and reduces the Hippocampal synaptic plasticity, which in turn leads to long-term learning and memory dysfunction (73). Therefore, regulating the level of BDNF has a certain guiding significance for the treatment of general anesthetic neurotoxicity and cognitive impairment in the developing brain.

## Other potential mechanisms

### Endoplasmic reticulum stress

Endoplasmic reticulum (ER) stress is a self-protective response of the body that induces apoptosis (74). Studies have found that ER stress mediates sevoflurane-induced neurotoxicity in the developing brain. Protein tyrosine phosphatase 1B (PTP1B) present in the ER membrane can regulate ER stress, and studies have shown that inhibition of PTP1B can alleviate sevoflurane-induced neurotoxicity in the developing brain and ultimately improve cognitive impairments with reduced learning, memory, and other abilities (75), therefore the development of general anesthetic neurotoxicity may be mediated by ER stress in the developing brain. And the regulation of PTP1B may be a potential treatment for general anesthetic neurotoxicity and cognitive impairment. One study found that isoflurane can induce neurotoxicity in larvae by causing ER stress through inhibition of mitochondrial function (76), and activation of mTOR can inhibit ER stress and the occurrence of this neurotoxicity (76, 77). Although not involved in the early brain development of mammals in this study, it is still instructive for the toxic effects induced by general anesthetics on developing nerves.

### Intracellular Ca^2+^

Intracellular Ca^2+^ overload can damage mitochondria and make them unable to produce ATP normally, eventually leading to apoptosis. Zhu et al. found that sevoflurane can increase intracellular Ca^2+^ to induce mitochondrial damage and mitochondria-mediated neuronal apoptosis (78). At the same time, studies have found that ketamine-induced neurotoxicity is related to N-methyl-D-aspartic acid (79), and Ca^2+^ influx also mediates neurotoxicity in the developing brain induced by isoflurane (80) and propofol (81). To sum up, the neurotoxicity of the developing brain induced by general anesthetics may be related to the increase of intracellular Ca^2+^, so inhibiting the influx of Ca^2+^may improve the nerve damage induced by general anesthetics. However, whether the Ca^2+^overload in the developing brain nerve cells caused by general anesthetics is related to the pathway that affects the apoptosis of nerve cells is not clear, which requires further exploration by researchers.

### DNA methylation

DNA methylation is a form of chemical modification of DNA that affects the plasticity of hippocampal synapses by regulating the transcription of related genes, thereby affecting cognitive function. Studies have found that the cognitive impairment caused by sevoflurane exposure in neonatal mice is related to the hypermethylation of hippocampal synaptic plasticity-related genes (82, 83). Many genes were hypermethylated in mouse hippocampal cell lines under isoflurane exposure (84). Cognitive impairment is associated with methylation modifications of a variety of genes, commonly including BDNF, the Reelin genes, the serine/threonine protein phosphatase 1 gene, and many others. The hypermethylated forms that appeared after these genes were modified all might affect the normal of cognitive function by affecting related signaling pathways such as Akt, leading to the reduction of dendritic spines in hippocampal neurons. Therefore, inhibiting the methylation of genes related to synaptic plasticity plays a vital role in improving the treatment of neurotoxicity and cognitive function caused by general anesthetics in the developing brain.

### Gut microbiota

The gut microbiota is a key regulator of brain development and function. Microbes in the gut associated with the brain's nervous system can affect the brain's cognitive function through the gut-brain axis (85, 86). General anesthetics can lead to dysbiosis in the gut microbiota of neonatal mice (87), and studies have shown that the pathogenesis of isoflurane-induced neurotoxicity in the developing brain may be related to the altered gut microbiota structure of juvenile rats caused by exposure (88). Recently, Wang et al. (89) found that the diversity and composition of their gut microbiota were significantly altered in juvenile rats prenatally exposed to isoflurane, and reduced BDNF expression was detected in the hippocampus. The imbalance of intestinal flora may be related to the pathogenesis of postpartum cognitive impairment in neonatal rats caused by maternal isoflurane exposure, which may be related to changes in immune response and increased susceptibility to infection. However, it is unknown whether the Akt signaling pathway is affected by the down-regulation of BDNF. It has been shown that differential abundances of cognition related microbial taxa were found in the gut of young rats exposed to sevoflurane for several times (90). The abnormal composition of these gut microbiota may be a risk factor for common general anesthetics induced neurotoxicity and cognitive impairment in the developing brain, but there are currently few studies on the causal relationship between gut microbiota and general anesthetics induced neurotoxicity and cognitive impairment in the developing brain, and whether it can mediate such neurotoxicity by inhibiting Akt related signaling pathways remains undefined and requires further investigation.

## Summary and outlook

The neurotoxicity and cognitive impairment of general anesthetics in the developing brain involve a variety of molecular mechanisms and signaling pathways, in which a variety of RNAs and related proteins directly or indirectly regulate the signal pathway of neurotoxicity induced by general anesthetics in the developing brain, and ultimately increase or reduce the neurotoxicity of this developing brain. By studying the related pathogenesis, we can further understand its potential signal transduction pathway, which is helpful for the selection of related therapeutic targets. Currently, some progress has been made in drug research on related targets, but the current drug research mainly focuses on the level of cells and animals, and there are few drugs used in clinical practice. Humans and animals differ in their brain structure and development, and the results of animal studies on the developing brain cannot be directly extrapolated to humans. Therefore, more in-depth research and related clinical trials should be conducted in the future to provide a new theoretical basis for the treatment of neurotoxicity and cognitive impairment caused by general anesthetic in the developing brain.

## Author contributions

JW: writing—original draft, validation, formal analysis, visualization, software, and methodology. ZL: methodology, writing—review and editing, funding acquisition, resources, supervision, and project administration. Both authors contributed to the article and approved the submitted version.

## Funding

This study was supported by the Inner Mongolia Medical University Joint Project (No. YKD2021LH063).

## Conflict of interest

The authors declare that the research was conducted in the absence of any commercial or financial relationships that could be construed as a potential conflict of interest.

## Publisher's note

All claims expressed in this article are solely those of the authors and do not necessarily represent those of their affiliated organizations, or those of the publisher, the editors and the reviewers. Any product that may be evaluated in this article, or claim that may be made by its manufacturer, is not guaranteed or endorsed by the publisher.
